# High-Throughput Sequencing Reveals the Gut Microbiome of the Bactrian Camel in Different Ages

**DOI:** 10.1007/s00284-019-01689-6

**Published:** 2019-04-27

**Authors:** Jing He, Le Hai, Khongorzul Orgoldol, Li Yi, Liang Ming, Fucheng Guo, Guowei Li, Rimutu Ji

**Affiliations:** 1 Key Laboratory of Dairy Biotechnology and Bioengineering, Ministry of Education, College of Food Science and Engineering, Inner Mongolia Agricultural University, Hohhot, 010018 Inner Mongolia China; 2 Camel Research Institute of Inner Mongolia, Alxa, 737300 Inner Mongolia China

## Abstract

The complex gut microbiota plays a key role in host metabolism and health. However, the core microbial communities in the different aged Bactrian camels remain totally unclear. We used high-throughput 16S rRNA gene sequencing to examine the temporal variability of the fecal microbiota in Bactrian camels. At 2 months of age, the fecal microbiota was composed of Firmicutes, Proteobacteria, and Actinobacteria. At 1 and 3 years of age, the fecal microbiota was dominated by Firmicutes, Bacteroidetes, and Verrucomicrobia. At the genus level, *Blautia, Fusobacterium,* and *Bifidobacterium* were more abundant at 2 months of age, as well as *Escherichia*–*Shigella*. *Ruminococcaceae_UCG*-*005, Akkermansia,* and *Christensenellaceae_R*-*7_group* were the most abundant at 1 and 3 years of age. Diversity and stability of the gut microbiota increased with age. There was enrichment for genes associated with immune system diseases at 2 months of age. This study is the first to investigate the distribution of the gut microbiota in Bactrian camels with different ages and provide a baseline for future camel microbiology research.

**Electronic supplementary material:**

The online version of this article (10.1007/s00284-019-01689-6) contains supplementary material, which is available to authorized users.

## Introduction

The establishment of a stable gut microbiota is closely correlated with host growth and immune development. The gut microbiota is an important factor for mammalian health, and plays a critical role in metabolism, immunity, and host development [1, 2]. A stable commensal community protects the host against invasive pathogens [3]. Microbial colonization of the infant begins at birth and is impacted by lactation [4, 5]. The gut microbiota in the ruminants not only regulates body health but also plays an important bridging role between diet and host [6]. It has demonstrated that ruminants have unique digestive properties and microbial which can help host to adapt to high fiber content foods, but also can make ruminants susceptible to a variety of diseases and conditions [7]. The gut microbiota in ruminants play a more prominent role in various physiological states [8].

The Bactrian camel inhabits the cold deserts of southern areas of central (Kazakhstan, Iran) and eastern (Russia, Mongolia, China) Asia [9]. The camel plays a vital role in the socio-economic of the region and is relied upon by millions of humans in both the semi-dry and arid areas. Previous studies also identified the species composition of the camel’s rumen microbiome [10] and a detailed profiling of the camel rumen’s carbohydrate-active enzymes [11]. Moreover, the gut microbiota may play a critical role in Bactrian camel health [12]. Microbial colonization begins at birth and is essential for the maintenance of host health. The composition of the early life microbiota has been studied in rats [13], cattle [14], and mice [15]. Furthermore, some potential links may also exist between age factors and intestinal microbiota. However, the dynamic shifts of the gut microbiota in Bactrian camels remain unclear. We used high-throughput 16S rRNA gene sequencing to investigate the microbiota composition of fecal samples from Bactrian camels. The results suggested that age is also important for shaping the Bactrian camel gut microbiota composition and contributing to its dynamic shifts.

## Materials and Methods

### Animals and Sample Collection

Samples were collected from 18 Bactrian camels in Inner Mongolia Bayannaoer in the spring of 2018. There were three groups: 2-month-old camels that were fed breast milk (TM); 1-year-old camels that were fed a plant-based diet consisting of Chenopodiaceae, Compositae, and Leguminosae (ON); and 3-year-old camels that were fed a plant-based diet consisting of Chenopodiaceae, Compositae, and Leguminosae (TH). Fresh fecal samples were immediately frozen using liquid nitrogen and were stored at − 80 °C. The experiment was conducted according to the animal ethics guidelines of the Key Laboratory of Dairy Biotechnology and Bioengineering, and approved by the Animal Ethics Committee of Inner Mongolia Agricultural University.

### DNA Extraction and 16S rRNA Gene Sequencing

Genomic DNA was extracted using the QIAamp DNA stool mini kit (QIAGEN, cat#51504), according to the manufacturer’s protocol. The V3–V4 hypervariable region of the 16S rRNA gene was amplified using primers the 515F and 806R primers (ACTCCTACGGGAGGCAGCA and GGACTACHVGGGTWTCTAAT, respectively). The reaction conditions were as follows: an initial denaturation at 98 °C for 2 min; followed by 30 cycles of denaturation at 98 °C for 15 s, annealing at 55 °C for 30 s, extension at 72 °C for 30 s; and a final extension at 72 °C for 5 min. The concentration and purity of DNA were tested using the Quant-iTTMPicoGreen^®^ dsDNA Assay Kit (Life Technologies, Grand Island, NY, USA). Library preparation and sequencing were conducted by the Personal Biotechnology Co., Ltd. (Shanghai, China).

### Data Analysis

The QIIME (Qiime1.8.0) was used for 16S rRNA data quality control and analysis [16]. We used the SILVA database (SILVA, Release 119) to analyze taxonomy [17]. The high-quality sequences were used in the final analysis. Sequences were clustered into operational taxonomic units (OTUs) using the UCLUST algorithm (97% similarity) in QIIME v.1.8.0. The Ribosomal Database Program (RDP) classifier was used to assign taxonomic category to all OTUs at a confidence threshold of 0.8. Alpha (Shannon, Chao1, Simpson, ACE), and beta (Bray–Curtis, weighted UniFrac) diversity metrics were calculated in QIIME. A one-way analysis of similarity (ANOSIM) was used to determine differences between groups. Differences between the TM groups and the two other groups were calculated using STAMP [18]. Differences in alpha diversity and relative abundance of taxa among different groups were analyzed using the Kruskal–Wallis rank-sum test in R. Differences between all three groups were determined using Welch’s test corrected for a false discovery rate (FDR) according to Benjamini–Hochberg procedure. PICRUSt v.1.0.1 was used to assess the metabolic potential of the gut microbiota [19].

## Results

### Microbiota Diversity

**Fig. 1:**
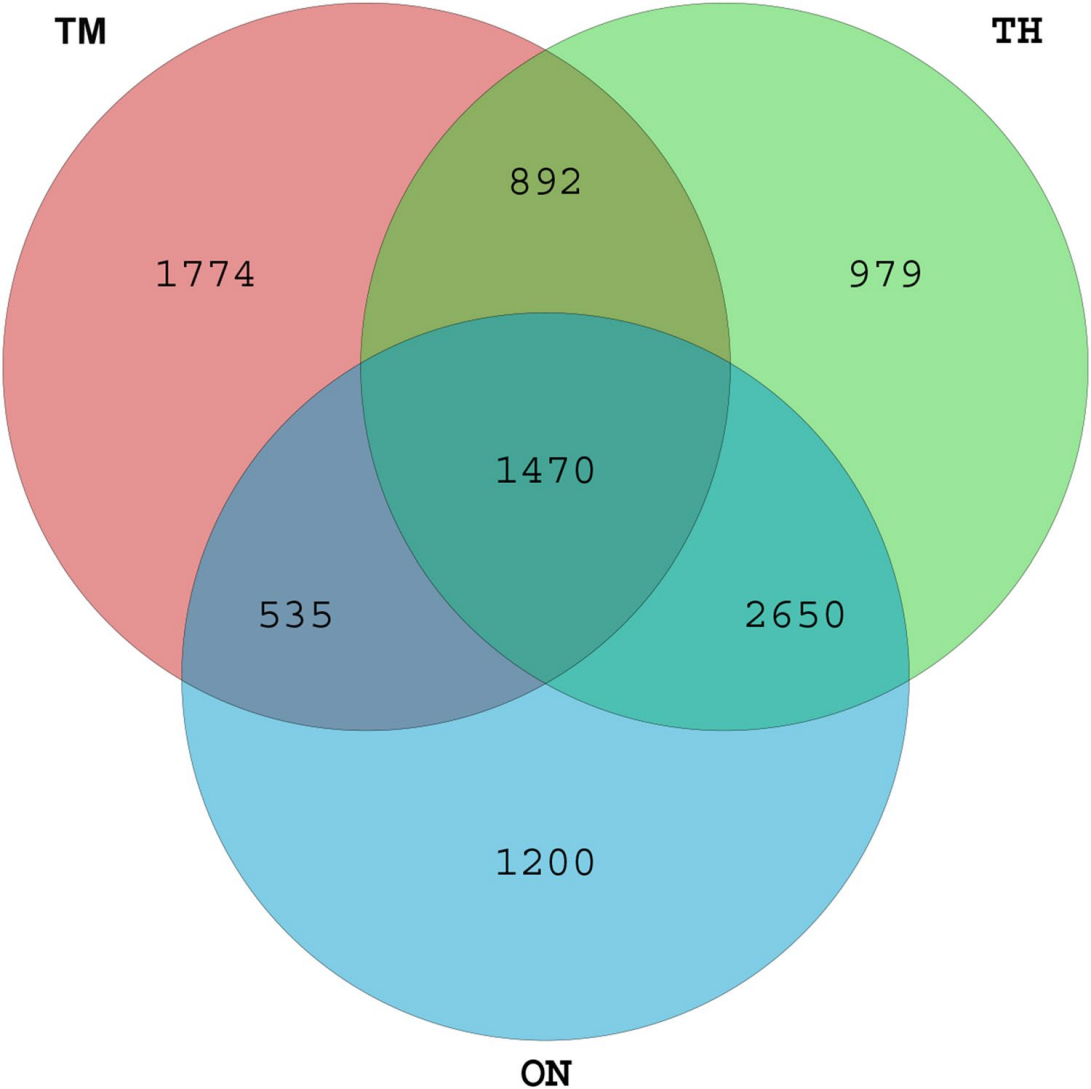
Venn diagram showing the overlap of OTUs across groups. *ON* 1 year old, *TH* 3 years old, *TM* 2 months old

We obtained a total of 766,114 high-quality sequences: between 33,247 and 47,873 valid sequences for each sample (Table S1). The rarefaction curves for the OTUs detected showed that the number of OTUs increased with the depth of sequencing. The final curve became stable, implying that the amount of sequencing data is somewhat reasonable (Fig. S1). Sequences were classified into 20 phyla, 43 classes, 67 orders, 113 families, and 234 genera (Table S2). There were 1470 OTUs shared by all three groups, and 1774, 949, and 1200 unique OTUs in the OM, TH, and ON groups, respectively (Fig. 1). The number of OTUs increased with age. The Prevotella, Butyrivirio, Clostridium.

**Fig. 2:**
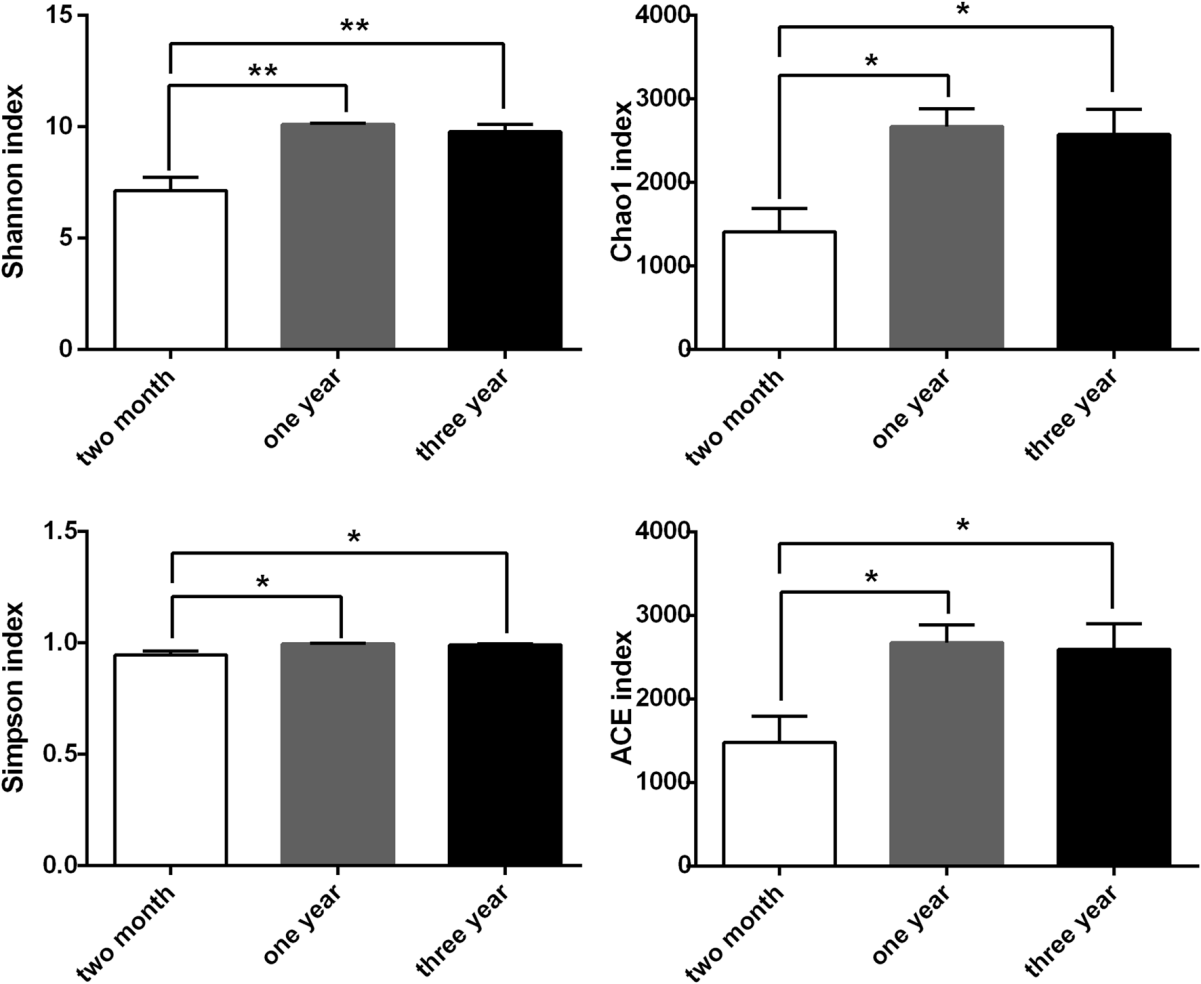
Differences in alpha diversity between the three groups. *P < 0.05, **P < 0.01

**Fig. 3:**
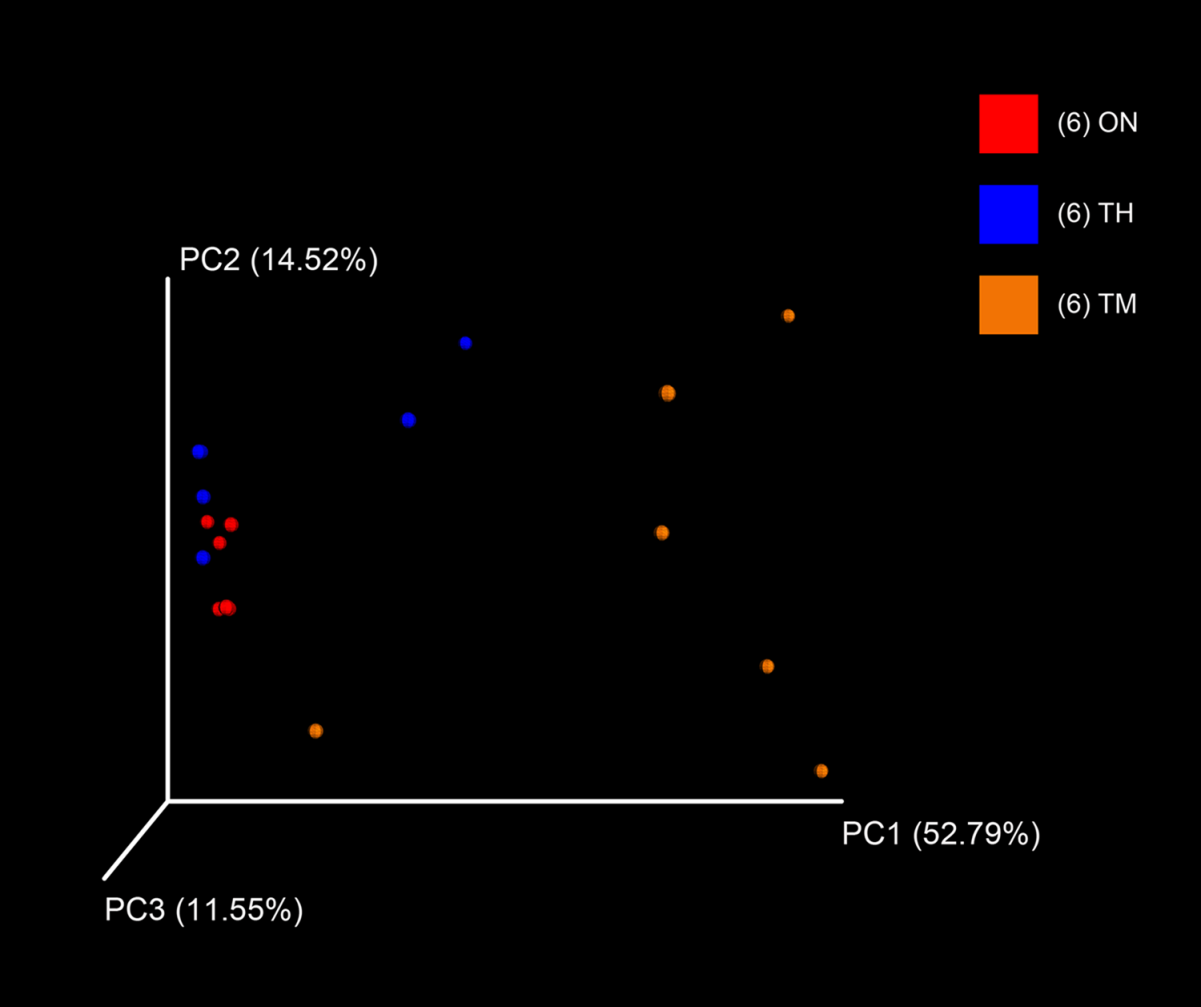
Principal coordinates analysis of weighted UniFrac distances. *ON* 1 year old, *TH* 3 years old, *TM* 2 months old

There were significant differences in alpha diversity (ACE, Chao1, Shannon, and Simpson indices) between the TM and ON groups (*P *< 0.05), as well as the TM and TH groups (*P *< 0.05) (Fig. 2). There was not a significant difference in the alpha diversity of the ON and TH groups (*P *> 0.05). A principal coordinates analysis of the weighted Unifrac distance showed that samples clustered according to age. The ON and TH samples were more similar than the TM samples with any other groups (Fig. 3). The composition difference was also determined by Partial Least Squares Discriminant analysis (Fig. S1) as well as analysis of similarities (ANOSIM) using weighted UniFrac distances (*R* = 0.5202, *P *= 0.001).

### Characterization of the Microbiota

**Fig. 4:**
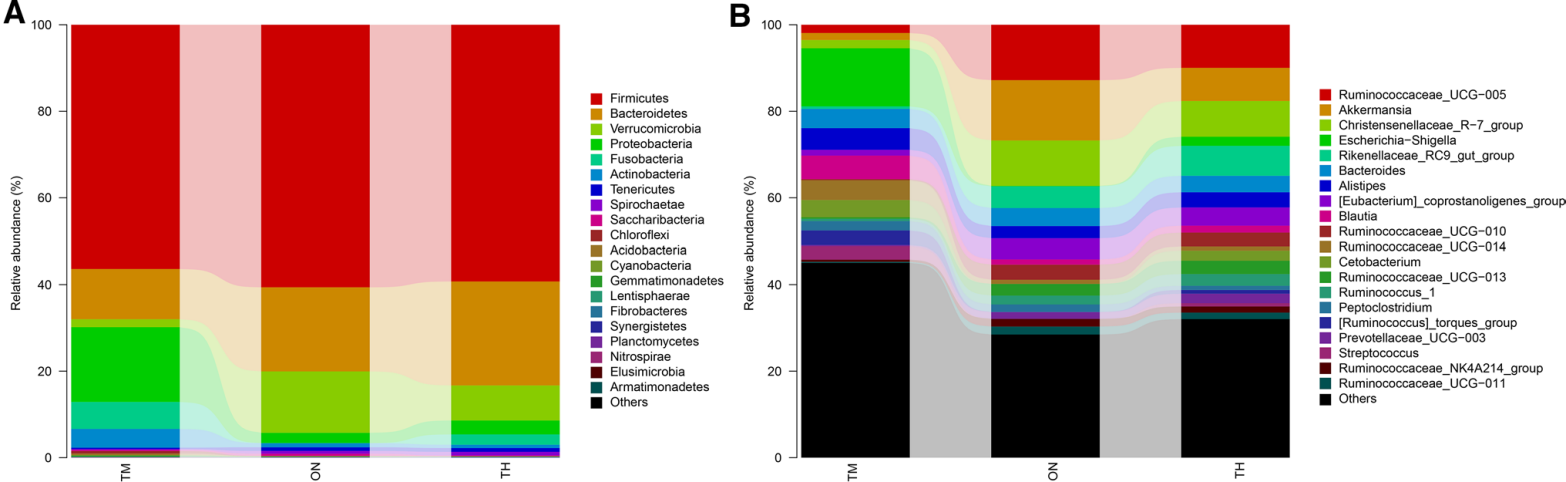
Microbial composition of different samples. **a** Taxa assignments at phylum level. **b** Taxa assignments at genus level. *ON* 1 year old, *TH* 3 years old, *TM* 2 months old

**Fig. 5:**
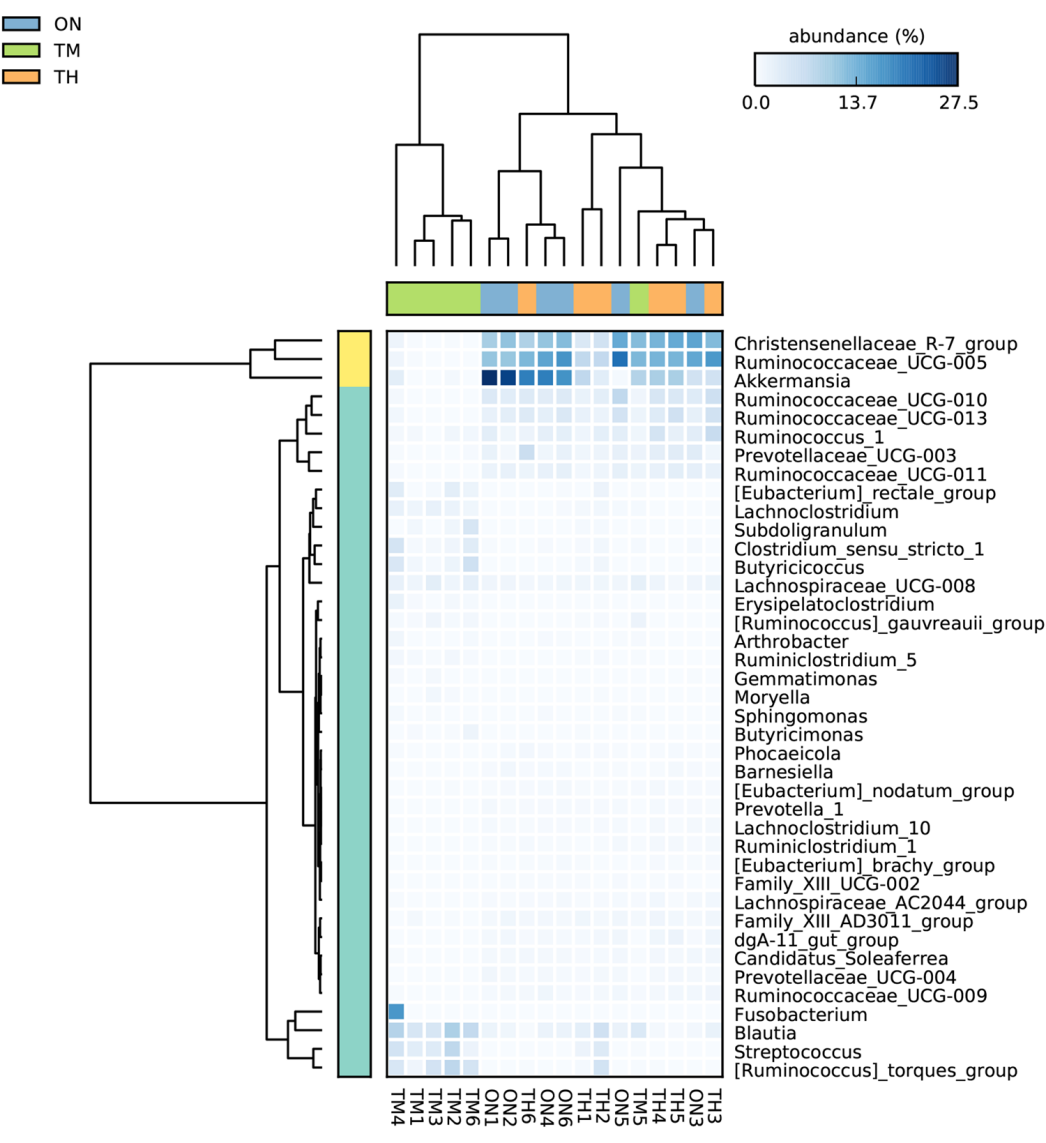
Heatmap of genus level relative abundances. *ON* 1 year old, *TH* 3 years old, *TM* 2 months old

**Fig. 6:**
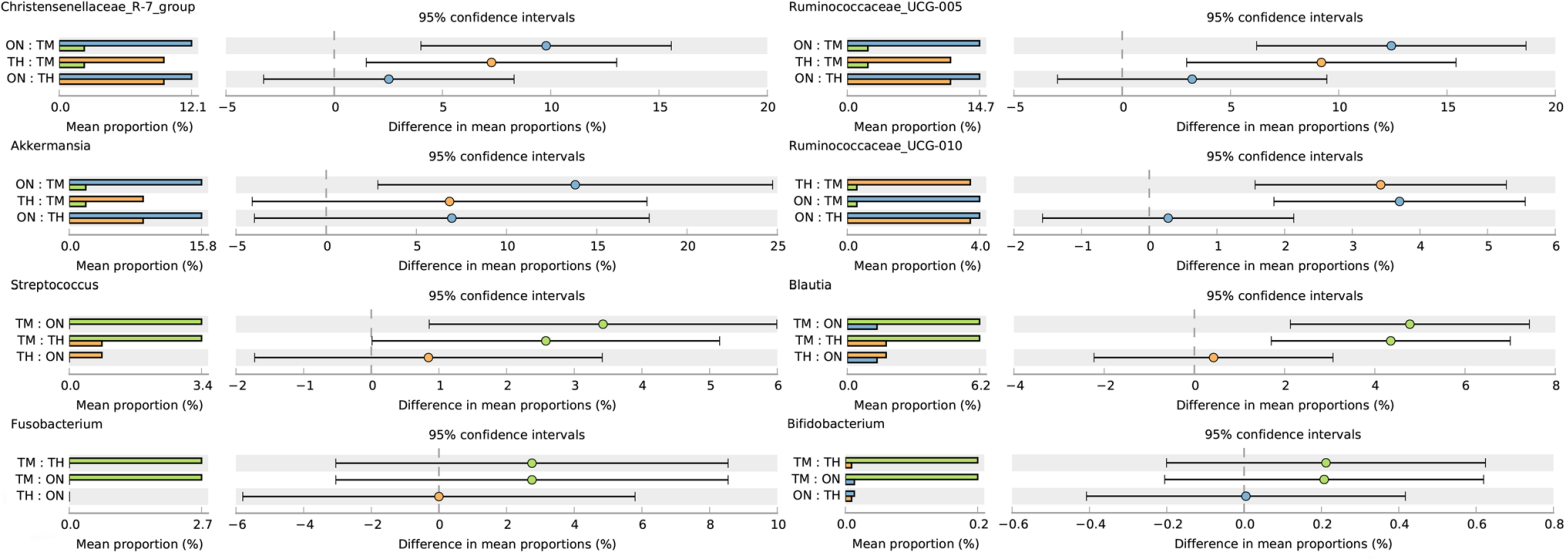
An extended error bar plot indicating the differences in mean abundance of taxa in the three groups. *ON* 1 year old, *TH* 3 years old, *TM* 2 months old

At the phylum level, Gut bacterial communities showed clear age differences in the Firmicutes (TH 59.27%, ON 60.62%, TM 56.39%), Bacteroidetes (TH 24.03%, 19.45%, TM 11.60%), Verrucomicrobia (TH 8.09%, ON 14.18%, TM 1.85%), and Proteobacteria (TH 3.22%, 2.39%, TM 17.28%) abundances (Fig. 4a, Table S3). The relative abundance ratios of Proteobacteria and Actinobacteria were higher in the 2-month-old group than other groups (*P *< 0.05). The microbiota of the ON and TH groups was dominated by Firmicutes, Bacteroidetes, and Verrucomicrobia (*P *< 0.05). At the genus level, the relative abundances of *Escherichia*–*Shigella* (13.45%)*, Blautia* (5.43%), and *Alistipes* (5.0%) were highest in the TM group; while *Ruminococcaceae_UCG*-*005* (ON 12.8%, TH 10.0%)*, Akkermansia* (ON 13.9%, TH 7.7%), and *Christensenellaceae_R*-*7_group* (ON 10.5%, TH 8.3%) were the most abundant in the TM and ON groups (Fig. 4b, Table S4). One sample in the TM group, TM5, had a markedly different community structure from the rest of the samples in that group (Fig. 5). Community structure in the ON group was similar to that of the TH group. In comparison with TM group, *Christensenellaceae_R*-*7_group, Ruminococcaceae_UCG*-*005, Ruminococcaceae_UCG*-*010, Akkermansia*, and *Prevotellaceae_UCG*-*003* increased significantly in the ON and TH groups (*P *< 0.05). However, The relative abundances of *Streptococcus, Blautia, Fusobacterium,* and *Bifidobacterium* were lower in the ON and TH groups than in the TM group (*P *< 0.05) (Fig. 6, Table S5).

### Predicted Functions of Microbiota

**Fig. 7:**
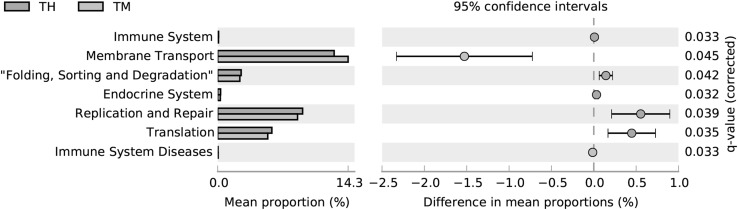
Predicted KEGG pathways for the TM and TH groups determined using PICRUSt. *TH* 3-year-old group, *TM* 2-month-old group

To estimate changes in the metabolic potential of the gut microbiota with age, we applied the PICRUSt algorithm. The metabolic capacities of the ON and TH groups were similar, while the TM group was distinct from the other two groups (Fig. S3). There were significant differences between the TM and TH groups in the predicted abundance of pathways related to immune system; folding, sorting, and degradation; replication and repair; and translation and immune system diseases (*P *< 0.05). The mean relative abundance of immune system disease-related pathways was higher in the TM group; while the relative abundance of immune system related pathways was lower in the TM group (Fig. 7).

## Discussion

Previous studies have described the longitudinal segregation of the gut microbiota in Bactrian camel [12]; however, the core gut microbiome of the Bactrian camel in different ages still remain unclear. To the best of our knowledge, in this study, we analyzed the bacterial diversity and abundance of fecal contents of Bactrian camel in different ages with high-throughput sequencing. In mammals, the distribution of microbes is influenced by host genotype, gender, and age [20, 21]. In this study, we show that the composition of the fecal microbiota changes during different ages. Firmicutes, Bacteroidetes revealed the most abundant taxa in the fecal microbiota. The abundance of these two major phyla was largely consistent with mature camel rumen [10]. At the genus level, *Prevotella, Butyrivirio*, *Spriochaetes, Verrucomicrobia, Succinivibria, Clostridium* were common in the core microbiome of camel rumen [10, 22]. However, the taxonomic groups represented within the Bactrian camel feces were not similar to those previously observed in the rumen of camels. *Ruminococcaceae_UCG*-*005, Akkermansia*, and *Christensenellaceae_R*-*7_group* were the most abundant in the mature fecal microbiota.

The richness and diversity of the microbiota increase with age [23, 24]. We observed differences in microbiota composition between the TM and ON/TH groups. By 1 year of age, the α-diversity (total OTU counts) approached adult levels and the β-diversity was similar to the TH group (Figs. 1, 2, 4). It is an indication that as Bactrian camel reached 1 year of age the microbiota gradually stabilized towards an adult-like state, which suggests that the 1-year-old Bactrian camel gut microbiome has many functional attributes of the adult microbiome.

Interestingly, at the phylum level, the increase in the relative abundance of Firmicutes, Bacteroidetes, and Verrucomicrobia that we observed, along with the decline in Proteobacteria and Actinobacteria and increase in Firmicutes and Bacteroidetes, is in agreement with previous studies [25, 26]. Microbiota diversity was primarily driven by diet. The dominant taxa in the TM group were Firmicutes and Proteobacteria, compared to Firmicutes, Bacteroidetes, and Verrucomicrobia for the ON and TH groups. Previous study has been reported that the Firmicutes/Bacteroidetes ratio changes with age [27]; Microbiota diversity was primarily driven by diet. The dominant taxa in the TM group were Firmicutes and Proteobacteria, compared to Firmicutes, Bacteroidetes, and Verrucomicrobia for the ON and TH groups. Previous studies have reported that the ratio of Firmicutes/Bacteroides changes with age [28], which is consistent with this report. Interestingly, the relative abundance ratios of Proteobacteria and Actinobacteria were higher in the TM group. Proteobacteria is one of the earliest colonizers and main members in the neonatal. It is conductive to homeostasis of the anaerobic environment of the GI tract gut [29]. Moreover, Actinobacteria coordination with one partner or host can easily be translated into pathogenic interactions with another [30].

At the genus level, *Escherichia*–*Shigella, Blautia*, and *Alistipes* were the abundant microbiota in the TM group. It has recently been reported that *Blautia* can exhibit beneficial anti-inflammatory effects [31]. Previous studies have described the dominance of *Escherichia*–*Shigella* in young calves and first-day human meconium [14, 24]. In addition to *Escherichia*–*Shigella*, *Streptococcus, Blautia*, and *Fusobacterium* had increased abundances in the TM group (*P *< 0.05) (Fig. 5). The proportion of some intestinal microbiota changes with age. Compared with camel calves, the proportion of the ratio of *Christensenellaceae_R*-*7_group, Ruminococcaceae_UCG*-*005, Ruminococcaceae_UCG*-*010,* were dominant in adult camels. *Ruminococcaceae* and *Christensenellaceae* regarded as potential beneficial bacteria because they participated in the positive regulation of the intestinal environment and linked to immunomodulation and healthy homeostasis [32, 33]. It suggests that health state is also related to ages. The significant differences for some KEGG categories, such as Immune system and immune system diseases, were also higher in the 2-month-old compared with other camels. The composition of the microbial community at 2 months of age may be heavily influenced by weaning, in which diarrhea is easy to occur. This shift from a milk- to a plant-based diet could also explain the differences in metabolic potential. The intestinal mucosal developmental immaturity and the external environment may make the camel calves more susceptible to pathogen invasion [34]. Previous study has showed that intestinal microflora was closely related to diarrhea [35].

In conclusion, our data suggest that microbiota composition may be due to changes in the diet and/or physiological changes associated with age. Gut microbiota structure is similar between the ON and TH groups, suggesting that maturation may occur by 1 year of age. However, we do not have enough time points to determine precise population dynamics. Bacterial colonization starts at birth and plays important roles in host growth and immune development [36]. A stable commensal community protects the host from invasive pathogens [3]. The establishment of stable microbial communities has an important role in the induction of homeostatic mechanisms [37]. Future work should examine the development of an adult microbiota as a basis for understanding how diet and host microbiota impact the development of the Bactrian camel.

## Electronic supplementary material

Below is the link to the electronic supplementary material.

Supplementary material 1 (DOCX 564 kb)

Supplementary material 2 (XLSX 14 kb)

Supplementary material 3 (XLSX 65 kb)

## Data Availability

Illumina sequence reads have been deposited under the NCBI SRA accession number SRP180948.
