# Supplementary material for: High-Throughput Sequencing Reveals the Gut Microbiome of the Bactrian Camel in Different Ages
Source: Curr Microbiol. 2019 Apr 27;76(7):810–7. doi: 10.1007/s00284-019-01689-6 (PMC6544607; doi:10.1007/s00284-019-01689-6)
Supplement: Supplementary file 1 — Supplementary material 1 (DOCX 564 kb) [file 284_2019_1689_MOESM1_ESM.docx]

Supplementary Material

**High‐throughput sequencing reveals the gut microbiome of the Bactrian camel in different age**

Jing He^1^, Le Hai^1^, Khongorzul Orgoldol^1^, Li Yi^1^, Liang Ming^1^, Fucheng Guo^1^, Guowei Li^1^ and Rimutu Ji^1,2^

^1^Key Laboratory of Dairy Biotechnology and Bioengineering, Ministry of Education, College of Food Science and Engineering, Inner Mongolia Agricultural University, Hohhot, 010018, Inner Mongolia, China

^2^Camel Research Institute of Inner Mongolia, Alxa, 737300, Inner Mongolia, China

**Table S1.** Read statistics for 16S rRNA gene sequencing. ON: one-year-old samples, TH: three-year-old Bactrian samples, TM: two-month-old samples.

| Sample | reads | Phylum | Class | Order | Family | Genus |
| --- | --- | --- | --- | --- | --- | --- |
| TM1 | 33247 | 734 | 734 | 734 | 717 | 633 |
| TM2 | 40823 | 1082 | 1082 | 1081 | 1073 | 972 |
| TM3 | 47167 | 752 | 741 | 684 | 655 | 545 |
| TM4 | 45598 | 1691 | 1667 | 1647 | 1606 | 1399 |
| TM5 | 47873 | 2151 | 2139 | 2124 | 2087 | 1875 |
| TM6 | 46423 | 1066 | 1058 | 1058 | 1049 | 932 |
| ON1 | 41503 | 2357 | 2356 | 2343 | 2290 | 2085 |
| ON2 | 43972 | 2408 | 2408 | 2405 | 2355 | 2177 |
| ON3 | 42805 | 2262 | 2262 | 2252 | 2200 | 1994 |
| ON4 | 46378 | 2777 | 2762 | 2753 | 2697 | 2440 |
| ON5 | 44303 | 2541 | 2528 | 2521 | 2478 | 2212 |
| ON6 | 40808 | 2429 | 2416 | 2407 | 2364 | 2152 |
| TH1 | 33279 | 1612 | 1612 | 1608 | 1584 | 1418 |
| TH2 | 39032 | 2065 | 2065 | 2060 | 2028 | 1831 |
| TH3 | 41411 | 2496 | 2496 | 2487 | 2456 | 2218 |
| TH4 | 40505 | 2419 | 2419 | 2408 | 2368 | 2115 |
| TH5 | 47318 | 2777 | 2777 | 2757 | 2724 | 2480 |
| TH6 | 43669 | 2487 | 2487 | 2478 | 2438 | 2199 |

**Table S2.** OTU statistics for 16S rRNA gene sequencing. ON: one-year-old samples, TH: three-year-old Bactrian samples, TM: two-month-old samples.

| Sample | Phylum | Class | Order | Family | Genus |
| --- | --- | --- | --- | --- | --- |
| TM1 | 9 | 17 | 21 | 35 | 93 |
| TM2 | 10 | 19 | 23 | 42 | 113 |
| TM3 | 18 | 43 | 52 | 75 | 114 |
| TM4 | 20 | 42 | 67 | 113 | 234 |
| TM5 | 16 | 38 | 58 | 92 | 195 |
| TM6 | 13 | 27 | 42 | 66 | 141 |
| ON1 | 17 | 32 | 54 | 79 | 145 |
| ON2 | 14 | 26 | 42 | 64 | 139 |
| ON3 | 16 | 27 | 40 | 65 | 139 |
| ON4 | 16 | 33 | 54 | 85 | 186 |
| ON5 | 18 | 34 | 47 | 77 | 176 |
| ON6 | 15 | 30 | 47 | 78 | 165 |
| TH1 | 14 | 25 | 31 | 50 | 126 |
| TH2 | 15 | 27 | 36 | 62 | 151 |
| TH3 | 12 | 21 | 27 | 47 | 116 |
| TH4 | 11 | 19 | 26 | 44 | 122 |
| TH5 | 14 | 22 | 34 | 52 | 130 |
| TH6 | 12 | 19 | 26 | 44 | 126 |

**Table S3.** The 16S rRNA copy number adjusted counts for phyla present in each sample. See Supplemental_Table_S3.csv.

**Table S4.** The 16S rRNA copy number adjusted counts for genera present in each sample. See Supplemental_Table_S4.csv.

**Table S5.** Comparisons of the relative abundance of genera (average relative abundance ≥5% in at least one GIT region) in the gastrointestinal tract of Bactrian camels using Kruskal-Wallis tests. Means in the same row with different superscript represent significant differences (P < 0.05).

| genus | phylum | TM | ON | TY | *P-*value | Corrected p-value |
| --- | --- | --- | --- | --- | --- | --- |
| *[Ruminococcus]_gauvreauii_group* | Firmicutes | 0.64 | 0.02 | 0.06 | 3.02e-3 | 0.030 |
| *[Eubacterium]_nodatum_group* | Firmicutes | 0.07 | 0.26 | 0.24 | 0.018 | 0.045 |
| *[Eubacterium]_brachy_group* | Firmicutes | 0.00 | 0.14 | 0.14 | 3.04e-3 | 0.025 |
| *[Ruminococcus]_torques_group* | Firmicutes | 3.36 | 0.04 | 0.87 | 2.34e-3 | 0.047 |
| *[Eubacterium]_rectale_group* | Firmicutes | 1.16 | 0.05 | 0.26 | 0.017 | 0.042 |
| *Arthrobacter* | Actinobacteria | 0.21 | 0.09 | 0.00 | 0.013 | 0.034 |
| *Akkermansia* | Verrucomicrobia | 1.63 | 13.94 | 7.66 | 0.021 | 0.048 |
| *Barnesiella* | Bacteroidetes | 0.02 | 0.33 | 0.17 | 2.91e-3 | 0.032 |
| *Blautia* | Firmicutes | 5.43 | 1.21 | 1.60 | 8.32e-3 | 0.031 |
| *Butyricicoccus* | Firmicutes | 1.80 | 0.00 | 0.19 | 0.010 | 0.033 |
| *Butyricimonas* | Bacteroidetes | 0.28 | 0.00 | 0.04 | 0.021 | 0.049 |
| *Candidatus_Soleaferrea* | Firmicutes | 0.08 | 0.51 | 0.51 | 3.40e-3 | 0.019 |
| *Christensenellaceae_R-7_group* | Firmicutes | 1.89 | 10.50 | 8.26 | 0.021 | 0.049 |
| *Clostridium_sensu_stricto_1* | Firmicutes | 1.48 | 0.14 | 0.18 | 0.011 | 0.034 |
| *dgA-11_gut_group* | Bacteroidetes | 0.02 | 0.51 | 0.73 | 2.49e-3 | 0.041 |
| *Erysipelatoclostridium* | Firmicutes | 0.43 | 0.00 | 0.05 | 9.10e-3 | 0.030 |
| *Family_XIII_AD3011_group* | Firmicutes | 0.19 | 0.60 | 0.72 | 6.06e-3 | 0.025 |
| *Family_XIII_UCG-002* | Firmicutes | 0.00 | 0.15 | 0.12 | 2.76e-3 | 0.035 |
| *Fusobacterium* | Fusobacteria | 2.14 | 0.00 | 0.00 | 8.54e-3 | 0.029 |
| *Gemmatimonas* | Gemmatimonadetes | 0.13 | 0.05 | 0.00 | 0.012 | 0.033 |
| *Lachnoclostridium* | Firmicutes | 1.21 | 0.11 | 0.25 | 6.58e-3 | 0.026 |
| *Lachnoclostridium_10* | Firmicutes | 0.01 | 0.28 | 0.24 | 3.04e-3 | 0.028 |
| *Lachnospiraceae_AC2044_group* | Firmicutes | 0.07 | 0.30 | 0.34 | 0.010 | 0.033 |
| *Lachnospiraceae_UCG-008* | Firmicutes | 1.72 | 0.89 | 0.90 | 8.41e-3 | 0.030 |
| *Moryella* | Firmicutes | 0.18 | 0.00 | 0.03 | 5.41e-3 | 0.024 |
| *Phocaeicola* | Bacteroidetes | 0.02 | 0.16 | 0.27 | 3.30e-3 | 0.021 |
| *Prevotella_1* | Bacteroidetes | 0.01 | 0.29 | 0.24 | 2.59e-3 | 0.037 |
| *Prevotellaceae_UCG-003* | Bacteroidetes | 0.28 | 1.56 | 2.19 | 0.015 | 0.037 |
| *Prevotellaceae_UCG-004* | Bacteroidetes | 0.03 | 0.48 | 0.46 | 4.97e-3 | 0.023 |
| *Ruminiclostridium_1* | Firmicutes | 0.03 | 020 | 0.30 | 0.013 | 0.033 |
| *Ruminiclostridium_5* | Firmicutes | 0.34 | 0.01 | 0.01 | 4.97e-3 | 0.023 |
| *Ruminococcaceae_UCG-005* | Firmicutes | 1.90 | 12.81 | 9.97 | 7.71e-3 | 0.030 |
| *Ruminococcaceae_UCG-009* | Firmicutes | 0.05 | 0.59 | 0.53 | 3.15e-3 | 0.024 |
| *Ruminococcaceae_UCG-010* | Firmicutes | 0.24 | 3.27 | 3.21 | 3.36e-3 | 0.020 |
| *Ruminococcaceae_UCG-011* | Firmicutes | 0.23 | 1.85 | 1.52 | 4.30e-3 | 0.023 |
| *Ruminococcaceae_UCG-013* | Firmicutes | 0.32 | 2.69 | 3.08 | 3.24e-3 | 0.022 |
| *Ruminococcus_1* | Firmicutes | 0.54 | 2.06 | 2.71 | 4.63e-3 | 0.023 |
| *Sphingomonas* | Proteobacteria | 0.09 | 0.12 | 0.00 | 0.011 | 0.032 |
| *Streptococcus* | Firmicutes | 3.03 | 0.00 | 0.75 | 4.85e-3 | 0.023 |
| *Subdoligranulum* | Firmicutes | 0.98 | 0.00 | 0.01 | 0.011 | 0.033 |


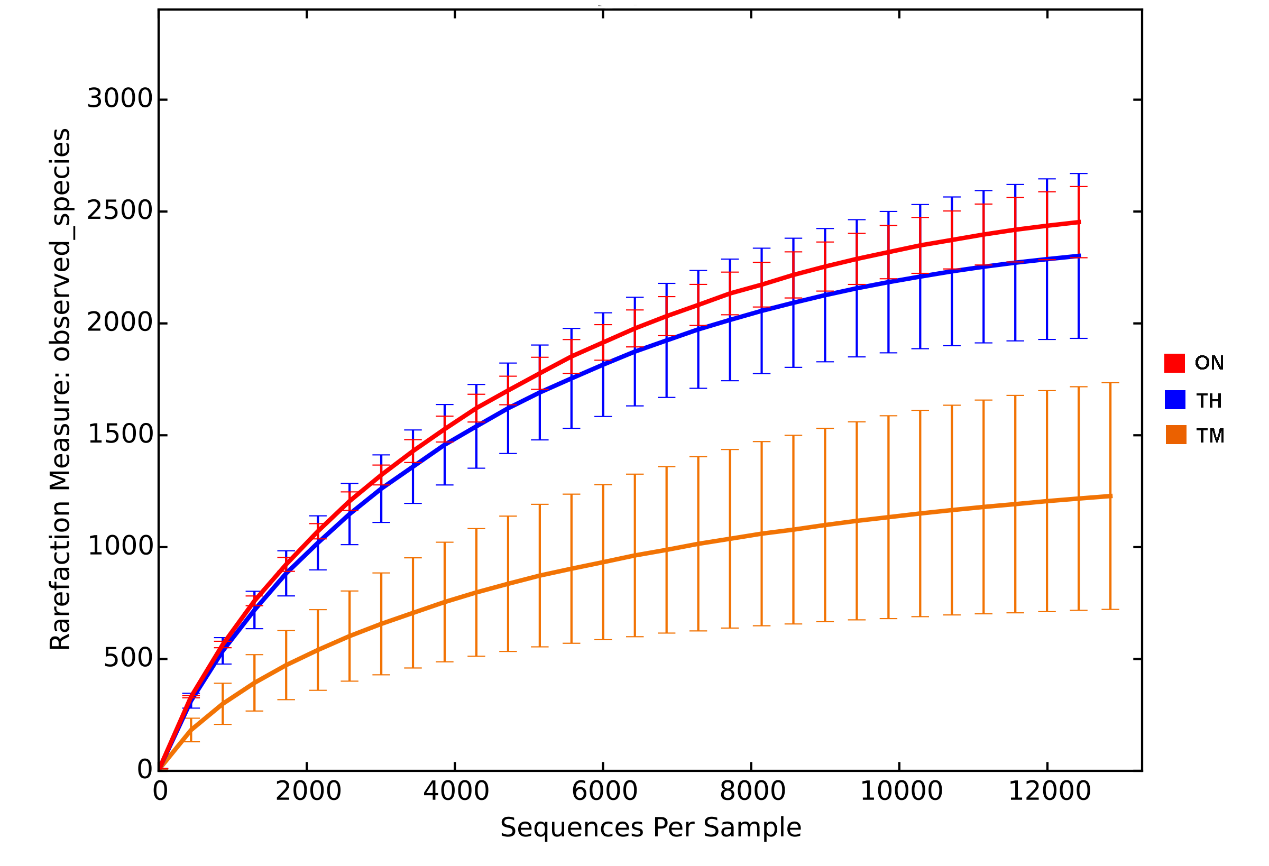


Fig S1 Summary of rarefaction results based on operational taxonomic units (OTUs) for each group. ON: one-year-old samples, TH: three-year-old Bactrian samples, TM: two-month-old samples.


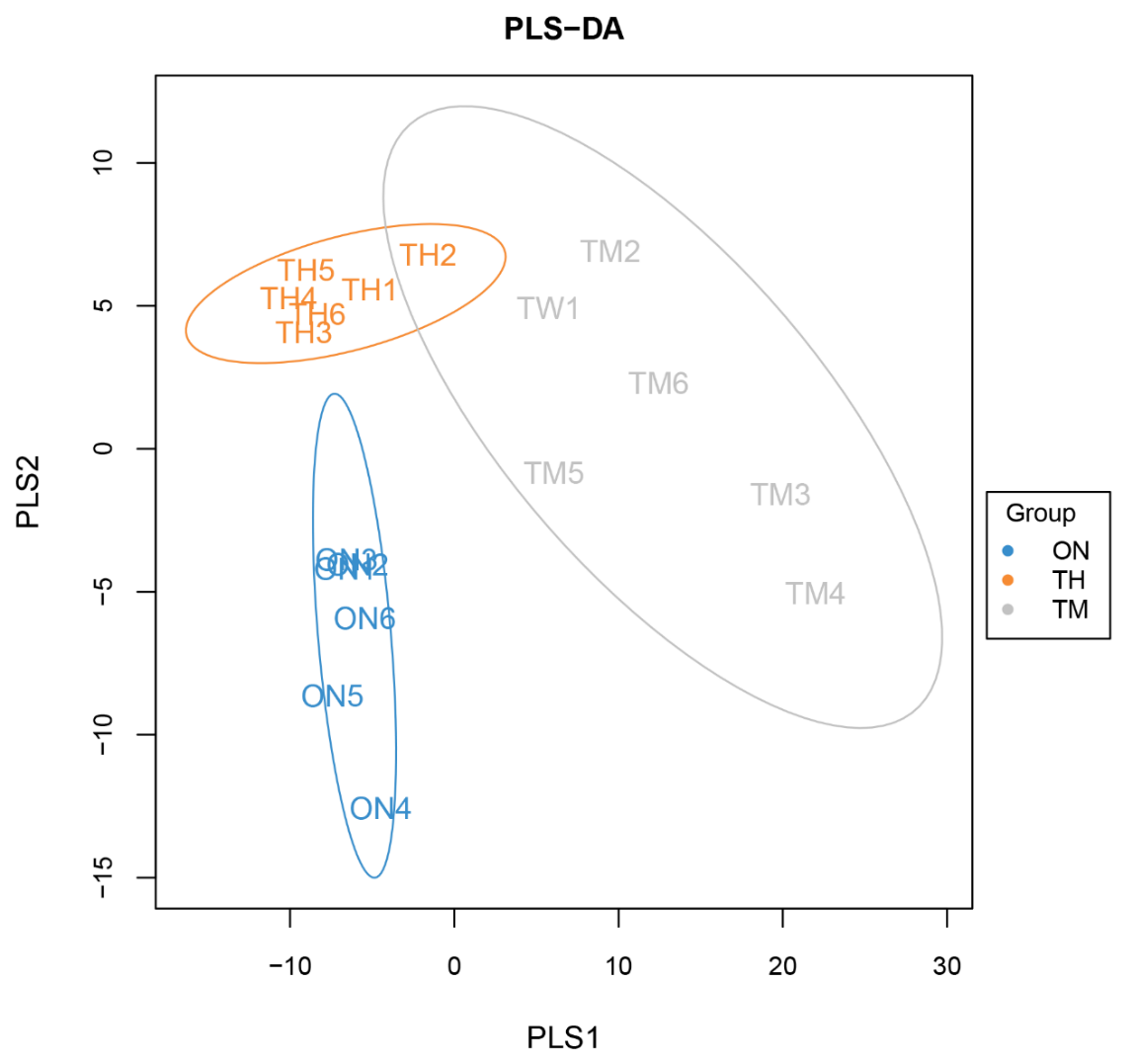


Fig S2 score plots showing the results of supervised partial least squares discriminant analysis with model fitness parameters. ON: one-year-old samples, TH: three-year-old Bactrian samples, TM: two-month-old samples.


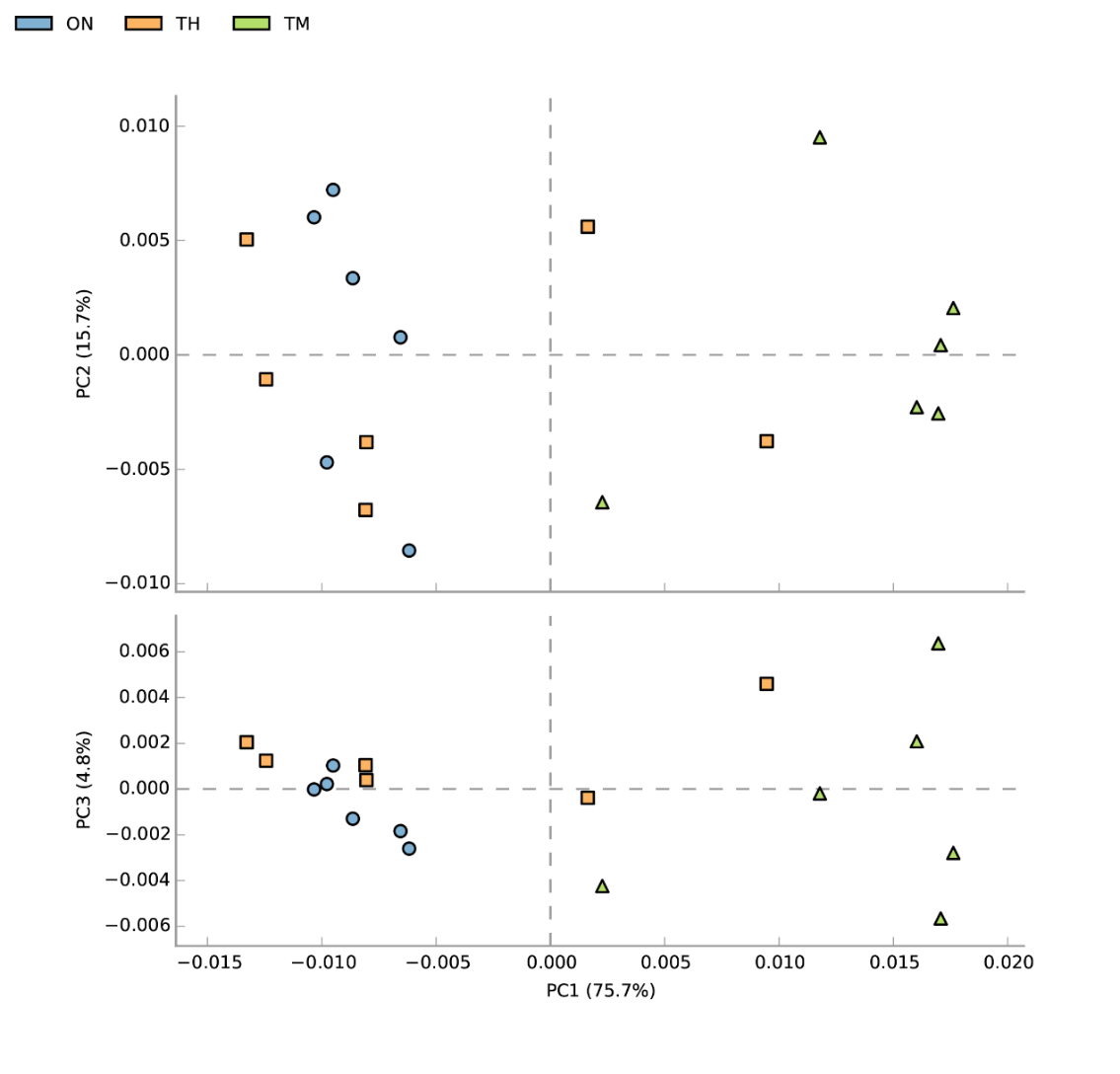


Fig S3. Predicted KEGG pathways obtained using PICRUSt for two-month-old and three-year-old Bactrian camels. PCA plot showing the differences among three groups for each KEGG Metabolism Pathway. ON: one-year-old samples, TH: three-year-old Bactrian samples, TM: two-month-old samples.
